# NEWS - RESOURCES

**DOI:** 10.7189/jogh.05.020203

**Published:** 2015-12

## DEMOGRAPHY

➢ After falling for 50 years, Egypt’s fertility rate has risen to 3.5 children per woman. This will almost inevitably cause faster population growth as infant mortality is falling and life expectancy is increasing. The population may rise to 140 million by 2050, who will live on the 5% of Egypt’s land which is habitable (along the river Nile and coastline). Egypt would be classified as “water poor” (ie, less than 1000 m^3^ of water per year) with a population higher than 55 million, and the country’s schools and hospitals would be increasingly overburdened. Birth rates tend to fall when people grow wealthier and women are better–educated, and Egypt’s rural poor have higher birth rates. The increasing number of births undermines Egypt’s demographic dividend–the economic advantage of having fewer old people and children relative to the number of working adults. “Meeting the demands of this population will require strong, sustained economic growth and redistributive policies,” according to Jaime Nadal Roig, head of the UN Population Fund’s Egyptian section. (*The Economist*, 6 June 2015)

➢ By 2050, 28% of the EU’s population will have reached retirement age, and the dependency ratio of over 65s to the economically–active 15–64 age group will increase from 27.8% to 50.1% by 2060. This could decrease Europe’s economic growth by 0.2% per annum, and would reach crisis point when pensions become unsustainable. Europe needs to rapidly increase its younger population to ward off this crisis, and increased migration is required to fully realise this. Enabling easier immigration by refugees seeking entry to Europe from eg, Syria, Iraq and Afghanistan makes economic and humanitarian sense, as they are mainly young people who are highly motivated to work. “Refugees are our future spouses, best friends or soulmates, the drummer for the band of our children, our next colleague, Miss Iceland in 2022, the carpenter who finally finished the bathroom, the cook in the cafeteria, the computer genius or the television host,” reads a petition from the Icelandic people to its government, urging more immigration. (*Bloomberg*, 4 Sept 2015)

➢ Tanzania has some of the lowest birth registration rates in eastern and southern Africa. 80% of Tanzanians – and more than 90% of children aged under 5 years–do not have birth certificates, according to the 2012 census. To help address this, Tanzania has launched a new system for birth registration, whereby health workers can send the baby’s name, gender, date of birth and family details by phone to a central database, and a birth certificate is issued free of charge. This side–steps the obstacles of distance and cost which prevented many parents from registering births. Birth registration is essential to ensure basic rights and access to health care, education and justice. It also protects against child labour, child marriage, trafficking and military recruitment. In recognition of this, the new Sustainable Development Goals includes a commitment to universal birth registration. (*Thomson Reuters Foundation*, 13 Oct 2015)

➢ China’s official media announced an end to the country’s 30–year–old one–child policy, reflecting growing concerns over the demographic problems of a shrinking workforce and an ageing population. This follows an easing of the policy in 2013, to allow couples who are themselves only children to have a second child. In 2012, China’s working–age population decreased for the first time in several decades, raising fears that China would be the world’s first country to age before it fully developed. However, only 12% of eligible couples had applied for permission to have a second child following the earlier relaxation of the policy, due to the high costs and difficulties in raising children in China. This may mean that ending the one–child rule could have limited effect on raising China’s birthrate. It was announced at the end of a meeting of the Communist Party leadership, designed to formulate policies to feed into a new “5 year plan” focusing on avoiding the “middle–income trap” and move from an investment and export developing country to a “moderately prosperous society” underpinned by services, consumer spending and innovation. (*Sydney Morning Herald*, 20 Oct 2015)

➢ In contrast to other age and ethnic groups–and in contrast to their counterparts in other developed countries–death rates amongst middle–aged, white Americans has risen. This is driven by high levels of suicide and alcohol– and drug–related causes, rather than eg, heart disease and diabetes. The mortality rate for this group (white, aged 45–54 years, high–school education or less) has increased by 134 deaths per 100 000 from 1999 to 2014. Death rates for middle–aged black people, Hispanics, and younger or older people fell in the same period. Increased use of opioid drugs and higher financial pessimism are part of the factors behind the increase. However, falling household incomes amongst this group, increasing difficulty in socialising and mobility problems are other contributing factors behind rising pain levels, poor health and distress. The USA has already fallen behind other developed countries in improvements in life expectancy. (*New York Times*, 2 Nov 2015)

## ECONOMY

➢ On face value, China’s economy seems highly innovative, as its annual spend of US$ 200 billion on research and development is the second–highest in the world, and it leads the world in patent applications and engineering graduates. Despite the headline figures, the economic impact of innovation is more muted, with decreasing contributions to economic growth rates. This must change in order to maintain China’s economic growth rates. China needs to improve innovation in customer–focused sectors, on improving efficiency; engineering new products and developing new products through the commercial application of basic scientific research. China must succeed at innovation in order to raise productivity and create high–value–added jobs–essential when wages rise and more workers migrate to the cities. (*McKinsey & Co*, Jul 2015)

➢ Since 1990, more than 1 billion people have escaped extreme poverty (living on less than US$ 1.25/d). However, 840 million people – 13% of the world’s population – still live in extreme poverty. Attention is now turning to eradicating extreme poverty by 2030. A panel convened by Brookings agree that the easier target of reducing poverty in stable, well–governed countries has been met. Attention must focus on tackling poverty in groups that may not be prioritised by their governments, and developing strategies than can work despite dysfunctional or weak governments. There are three key challenges in overcoming extreme poverty; peace, jobs and resilience. Peace is a pre–requisite and a result of poverty elimination, and people must have basic level of security in order to prosper. Jobs and sustainable incomes are essential to ending poverty; and job growth needs infrastructure, the rule of law, property rights etc. Resilience, which provides a safety net to cope with unexpected expenses or events, can be provided by eg, insurance against crop failure. Some experts note that economic growth alone cannot end extreme poverty. Ana Revanga of the World Bank calls for redistribution of wealth and equity, and also emphasised agricultural development, and access to health and education. (*Brookings*, 27 Jul 2015)

➢ The EU, one of the world’s richest and most peaceful regions, has taken far fewer of the asylum–seekers fleeing Islamic State compared to other countries such as Lebanon. The EU also compares badly to Tanzania, which has hosted hundreds of thousands of Congolese and Burundian refugees. There are strong economic grounds for Europe hosting more asylum–seekers, as well as humanitarian reasons. Europe faces an ageing labour force and high levels of government debt that future generations will struggle to repay. Migrants, including asylum–seekers, are normally young and keen to work; this would ease the burden of an ageing population and indebtedness. Globally, migrants are net contributors with lower crime rates and higher likelihoods of starting businesses. And if labour markets reforms make it easier for migrants to work, it will support closer integration and make it less likely that young unemployed men are attracted to extremist groups. Europe could emulate the USA’s economic success which was built on earlier immigration. The message is: let them in – and let them earn. (*The Economist*, 29 Aug 2015)

➢ The IMF warned that the economic slowdown in emerging markets is affecting global economic growth, which is at its lowest level since the financial crisis. There is an increasing risk of a global recession, and the IMF revised its estimate for 2015 global economic growth downwards, from 3.3% to 3.1%. It also cut its estimate for 2016 to 3.6%. Investors are pulling out funds from emerging markets, and it is estimated that 2015 will see the first net capital exodus for 27 years, with more than US$ 1 trillion leaving countries like Brazil, Turkey and South Africa. Although China’s booming service sector off–sets its slowing manufacturing sector, there are external negative effects on global commodities suppliers which are reliant on Chinese markets. The outlook is worsened by emerging economies’ increased debt levels – often in US dollars, and hence more vulnerable to rising US interest rates – and higher default risk. In response, the IMF has called for the US Federal Reserve to delay its planned rise in interest rate, for Europe to deal with its trillions of US dollars of bad debt, and for emerging markets to overhaul their economies and strengthen their resistance to financial risk. (*Wall Street Journal*, 8 Oct 2015)

➢ Sub–Saharan Africa is home to many of the world’s fastest–growing economies, with increasing interest from international private equity investors seeking to benefit from the continent’s economic growth and emerging middle–class consumers. A report from the UK’s Overseas Development Institute (ODI), shows that this investment has grown 500% since 2008 and accounts for US$ 12 billion – or 20%–of annual cross–border investments. Capital flow is essential for economic development, but the continent still suffers from a lack of investment opportunities, with firms often being too small and lacking in human capital. The ODI calls for development finance institutions to support the creation of more medium–sized businesses, and to provide investors with less costly and more flexible risk insurance tools. (*Reuters*, 8 Oct 2015)

## ENERGY

➢ According to the World Bank, power shortages reduce economic growth by up to 4% each year in sub–Saharan Africa, with many businesses forced to rely on expensive generators. Poorer people are disproportionately affected, as they spend up to 16% of income on energy, and use expensive kerosene or batteries for cooking and light. The capacity installed by independent power producers in Africa has grown by 14% each year since 1992–and an ever–increasing share is going to renewable energy. This is partly because Africa has some of the world’s best untapped resources for renewable energy (eg, rivers and hot deserts), and renewable energy sources can be set up quickly and cheaply to meet shortages. It is easier to connect remote villages to a local renewable energy source than a main grid. With falling equipment prices, and the right regulatory environment and access to finance, Africa could become a leading producer of clean energy – making it richer and greener. (*The Economist*, 6 June 2015)

➢ In the past decade, more than US$ 2 trillion has been invested in renewable energy. According to the International Energy Authority, cumulative investment in low–carbon energy supply and energy efficiency must reach US$ 53 trillion by 2035 to keep global warming within the 2°C safety limit. This figure sounds daunting, but it is only 10% higher than the energy investment required (US$ 53 trillion) to keep apace with economic and population growth. The price difference between conventional and renewable energy is narrowing, and clean energy could support the transition to a low–carbon economy and meeting economic and development goals. However, national governments’ local content requirements (LCRs), which normally require solar or wind power developers to source a given percentage of jobs, supplies or resources locally are hindering international investment in these energy sources. LCRs increase the cost of intermediate inputs, and lead to less competition, therefore deterring investment. The OECD calls for multi–lateral co–operation to address these barriers to trade and investment in clean energy. (*OECD*, 9 June 2015)

➢ Since 2012, Iran’s government has pushed renewable energy as an alternative to fossil fuels, which currently supply 94% of its electricity. Energy developers are building wind turbines on ridges, and Iran aims to install 5 gigawatts of renewable energy capacity by 2020, making it comparable to France and the UK. The prospect of sanctions being lifted after the signing of the nuclear deal on 14 July has led to increased interest in all energy developments, with oil companies keen to exploit Iran’s oil fields, which need an estimated US$ 200 billion of investment. Banks are also willing to start lending again, once payment systems are restored and sanctions are lifted. However, some developers are pushing ahead with financing, such as Umwelftconsult, who are seeking US$ 44 million for the installation of wind turbines to generate 47.3 megawatts of electricity – and more will follow. (*bloomberg.com*, 30 Jul 2015)

➢ The oil and gas company Statoil will build the world’s largest floating offshore windfarm, off the northeastern coast of Scotland. The project (Hywind Scotland) differs from conventional offshore windfarms by using turbines attached to the seabed by a mooring spread and anchoring system, and could power nearly 20 000 homes. Floating wind farms may reduce generating costs for offshore developments to under US$ 150MWh, with larger projects reducing costs to US$ 130–140MWh. Currently, the average global price is US$ 170MWh. A report from the Energy Technologies Institute (ETI) suggests that offshore wind could be a cost–effective form of low–carbon energy for the UK by 2025. Ms Irene Rummelhoff, Statoil’s VP for new energy solutions says “floating wind represents a new, significant and increasingly competitive renewable energy source. Statoil’s objective with developing this pilot park is to demonstrate a commercial, utility–scale floating wind solution, to further increase the global market potential.” (*The Guardian*, 2 Nov 2015)

➢ South Africa proposes to build 8 new nuclear power stations, at a cost of R1 trillion (US$ 72 billion). Expanding South Africa’s energy capacity is essential for economic growth and housing development, and the country suffers from power outages and ageing infrastructure. South Africa, like other countries after the Fukushima nuclear disaster, had considered delaying the expansion of its nuclear energy capacity. However, the government appears to have climbed down over this policy, with its surprise announcement of a large deal to build new nuclear power stations, with Russia as the preferred supplier. This move is subject to intense criticism over its speed and lack of transparency, and there has been allegations of corruption. There is scope for covering the country’s energy shortfall by increased investment in renewable energy and energy saving strategies. (*theconversation.com*, 5 Nov 2015)

## ENVIRONMENT

➢ Irrigation and the increased watering of fields in sub–Saharan Africa are creating conditions ideal for invasive plant pests, such as the tomato leaf miner. This moth attacks crops like tomatoes, potatoes, peppers, aubergines and tobacco. This insect is native to South America, but spread to Africa via Europe. East Africa is particularly vulnerable, as increased rainfall and temperatures as a result of climate change–combined with irrigation – creates warm and damp environments ideal for crop–eating insects from tropical climates. According to Dan Bebber from the University of Exeter, UK, this poses dangers for food production. “Irrigation could trigger changing pest distributions by allowing a host plant to grow where it would not otherwise growth, and by producing conditions for the pest or pathogen to grow. Also, pathogens can be spread around in irrigation water on a small scale.” The International Maize and Wheat Improvement Center in Kenya is trying to alleviate the problem by training farmers to introduce natural predators to manage the pest. (*scidev.net*, 13 Aug 2015)

➢ In 2010, Brazil passed a law which required all that solid waste be deposited in modern landfills, lined to prevent toxins from soaking into the soil. This has failed to stop rubbish being dumped unsafely, caused by a shortage of money and the political will to enforce legislation. The problem of unsafe dumping is worse in poorer areas with fewer landfill sites and less money. 60% of Brazil’s municipalities failed to meet these requirements, but none have been sanctioned. Moreover, the federal district around Brazil’s capital city, Brasilia, continues to dump its waste in unregulated sites 15km from the city centre. Fortunately, the main transport routes used by the country’s law–makers in the capital remain pristine. (*The Economist*, 15 Aug 2015)

➢ Vietnam is increasingly reliant on coal–fired electricity, with demand growing at one of the highest rates in South–east Asia, supporting one of the world’s fastest–growing economies. Demand is predicted to grow by 10–20% each year up to 2030, and 50% of energy will be generated by coal by 2020 – currently 33%. This has serious implications for air quality, and the WHO has linked air pollution from solid fuels to 3.7 million deaths – 70% in Asia and the Pacific region. Vietnamese mines or coal power stations do not take account of climate change or related extreme weather, and recently 17 people died in floods in north–eastern Vietnam which were linked to extreme weather events. “This is the first environmental disaster in Vietnam’s coal–mining industry, and the consequences are yet to be identified in terms of scale, duration and intensity. It’s time for Vietnam to evaluate comprehensively the national energy security related to power sources,” says Nguyen Dang Anh Thi, of the World Bank’s International Finance Corp’s resources efficiency programme. (*bloomberg.com*, 17 Aug 2015)

➢ Indonesia’s forest and peatland fires have killed at least 19 people, and 500 000 people have suffered from severe respiratory illnesses from the resulting haze. Across Southeast Asia, an estimated 110 000 people die each year from smoked caused by forest fires, mainly due to heart and lung problems. These fires are a man–made crisis, with most danger spots arising from oil palm and pulp wood plantations. The current haze is largely arising from dry and deforested peatland, as peat swamps are naturally resistance to fire, but are highly flammable when they are dried out and degraded. In response, President Joko Widodo has instructed the Forestry and Environment Ministry not to issue new permits for peatland monoculture cultivation, and to restore damaged peatland and review all existing peatland licences. (*Jakarta Post*, 1 Nov 2015)

French economists Lucas Chancel and Thomas Piketty have proposed a global US$ 196 levy on business class air tickets, and US$ 21 to raise US$ 150 billion needed each year for climate adaptation. Currently, according to the OECD only 16% of climate finance is targeted at adaptation – the remainder funds low carbon projects. This proposal would use air travel as a proxy for affluence across countries, as the wealthiest elite in developing countries are beginning to outstrip working–class Europeans in carbon emissions. There are huge disparities in carbon footprints, with 10% of people being responsible for 45% of global emissions. “Taxing flights is one way to target high–emitting lifestyles, especially if we tax business class more than economy class,” says Lucas Chancel. (*Climate Change News*, 5 Nov 2015)

## FOOD, WATER AND SANITATION

➢ 25% of the world’s food is wasted due to inefficient harvesting, inadequate storage and domestic wastage. If this were halved, an additional billion people could be fed–particularly crucial when 1–in–9 people (795 million in total) go hungry each day. In developing countries most wastage occurs at the production and storage stages. A lack of infrastructure is part of the problem – eg, inadequate roads make it harder for farmers to sell their surplus, and more reliable electricity supplies would enable grains to be dried and vegetables to be kept cool. It would cost an estimated US$ 239 billion to halve post–harvest losses in the developing world by investing in infrastructure, generating US$ 3 trillion of benefits. This would make food more affordable, and 57 million people would no longer be at risk of hunger. However, investing in improved food production, targeted at the major food crops and including small farmers would engender greater benefits. An additional US$ 88 billion investment each year would increasing yields by 0.4% – giving additional benefits of US$ 3 trillion. (*The Guardian*, 24 Jun 2015)

➢ A study published in *PLoS Medicine* suggests that pregnant women who defecate in the open are more likely to have a premature delivery or give birth to a baby with low weight than those who use toilets. India has the highest number of premature births in the world at 3.5 million (followed by China at 1.17 million), and nearly half of India’s population defecate in the open. Premature births and low birthweight are linked to health problems up to adulthood, including diabetes, hypertension and depression. The study also concluded that higher levels of education are associated with reduced risks of adverse pregnancy outcomes. India’s Prime Minister, Narendra Modi, has pledged to improve India’s sanitation, and wants every home to have a toilet by 2019. (*BBC*, 8 Jul 2015)

➢ 2.1 billion people – 30% of the world’s population – are overweight. This is double the number in 1980, and more than 2.5 times higher than the number of chronically–hungry people. According to a report by the McKinsey Global Institute, being overweight or obese is linked to 2.8 million deaths annually due to conditions like diabetes, cancer and cardiovascular disease. Developing countries have 30% more overweight and obese children compared to developed countries. It estimates the annual cost as US$ 2 trillion – 2.8% of global GDP. In light of this, the WHO cuts its recommendation for sugar consumption from 10% to 5% of adult daily calories. The report offers several other recommendations, eg, smaller fast–food portions, advertising restrictions, better nutritional information, reformulating processed foods, more exercise at school, and healthier meals in the workplace and school. These interventions, although difficult to achieve, do work; this is shown by the 43% decrease in childhood obesity in the USA in the past 10 years. (*Project Syndicate*, 20 Jul 2015)

➢ According to the latest Global Hunger Index despite population growth, deaths from large–scale hunger have fallen from 1.4 million in 1970 to 40 000 a year since 2000. This is accompanied by hunger levels falling by 30% since 2000. However, hunger is still a critical issues, with 800 million people being chronically undernourished. The Index also shows countries with the most improved records in fighting hunger (eg, Ukraine, Brazil and Mongolia), and the least improved, eg, Chad and Iraq. China has witnessed spectacular improvements in food security, changing from having 50% of the world’s deaths from famine to none. There are several countries with “alarming” hunger levels, including those experiencing conflict and epidemics which undermine food security. The UN’s Food and Agricultural Organization also notes the difficulties in generating estimates for countries with unreliable data, and are trying to monitor food security with measures that are less reliant on government stability, noting that deliberate starvation is still used as a tactic of war. (*NPR Goats and Soda*, 16 Oct 2015)

➢ The aid agency Goal warns that Ethiopia is facing its worst drought in decades, and more than 8 million people are in need of food assistance. This is greater than the 2011 drought in the Horn of Africa, which claimed 200 000 lives in Somalia. More than 80% of Ethiopia’s people work in agriculture, rendering the country particularly vulnerable to drought and climate change, and food prices are already increasing. The UN Office for the Co–ordination of Humanitarian Affairs (OCHA) estimates that 15 million people will need food assistance–more than Syria–350 000 people will suffer from severe acute malnutrition, 450 000 livestock deaths which would destroy livelihoods and increase food insecurity; and 1.8 million people would be affected by drinking water shortages. The Ethiopian government has diverted US$ 200 million from infrastructure expenditure into food supplies, and has appealed for funds. The OCHA estimates that US$ 451 million will be required. “The concern is that if there is not a timely response the situation will deteriorate,” says John Rynne, Goal’s director in Ethiopia. (*Irish Times*, 30 Oct 2015)

## PEACE AND HUMAN RIGHTS

➢ The World Justice Project’s index on corruption and justice, which measures the rule of law in 102 countries, is based on responses from 1000 citizens in each country. Its indicators are: constraints on government power; absence of corruption; open government; fundamental rights; order and security; regulatory enforcement; civil justice; criminal justice; and informal justice. It ranks Denmark at the least corrupt country, and Venezuela as the most corrupt. The results will inform discussion on the post–2015 development agenda, which emphasises good governance and rule of law as underpinning economic and social progress. The sustainable development goals will include commitments to promoting the rule of law, ensuring access to justice, end corruption, and ensure transparent and accountable institutions. (*The Guardian*, 2 Jun 2015)

➢ Myanmar’s Muslim Rohingya population have been described as”the most persecuted minority in the world”. They cannot claim citizenship in Myanmar, or other countries. They are fleeing repression and brutality in Myanmar, with an estimated 100 000 Rohingyas estimated to be in Malaysia (where they have no legal status and forbidden to work), and hundreds of thousands have thought to have fled to Bangladesh (where, in an UN–supervised operation, 200 000 Rohingyas were brutally repatriated in the 1990s). In Myanmar, no–one has faced prosecution or imprisonment for the attacks and murders on the Rohingya population in 2012. Human rights campaigners warn that they are “at grave risk of additional mass atrocities and even genocide”, noting that some of the pre–conditions of genocide (stigmatisation, harassment, isolation and the weakening of civil rights) are already in place. (*The Economist*, 13 June 2015)

➢ Nine Islamic countries have stoning as a judicial sentence, and five have amputation as judicial sentences. This is despite Islam’s sacred texts prescribing less harsh punishments than Judaism or Christianity, and allowing for the forgiveness, and hence sparing, of murderers. Under the Ottoman empire, one person was stoned to death in 600 years, but since the 1970s ever–harsher punishments have been introduced, with Iran, Pakistan and Afghanistan implementing sharia law. Islamic law definitions are being stretched to include homosexuality and apostasy as punishable offences. This is possible because Islamic law does not rely solely on the Koran as a law source, but includes the recorded sayings and actions of the Prophet Muhammad. However, the main reason is the instability of the Islamic world, where punishments can be used to target opposition groups, and placate hardline clerics or public opinion. Even when punishments are eased (eg, stoning in Iran), this tends to be via moratorium rather than abolition. Liberal lawyers in Saudi Arabia are campaigning for penalties to be codified to prevent harsher sentences when alternatives are available, although the fact that the regime’s jurisprudence says that lashes, stoning and the death penalty are necessary is inescapable. (*The Economist*, 4 Jul 2015)

➢ Amnesty International, the human rights campaigning group, has voted to support a policy that calls for the full decriminalisation of all aspects of consensual sex work. This was in the face of intensive lobbying from opponents who are opposed to exempting buyers and managers from penalties. Amnesty International argues that decriminalisation is the best way to reduce risk for sex workers, who can face arbitrary arrest and detention, extortion, harassment, and physical and sexual violence. Following the vote, Amnesty International will develop a final policy that can be used to lobby governments to repeal most laws that forbid the sale and purchase of sex. However, Amnesty International does not plan to have a major, global campaign on decriminalisation. Instead, national branches will take forward their own campaigns. (*New York Times*, 11 Aug 2015)

➢ Tunisia’s National Dialogue Quartet – a coalition of union leaders, business people, lawyers and human rights activities–won the 2015 Nobel Peace Prize for “its decisive contribution to the building of a pluralistic democracy in Tunisia in the wake of the Jasmine Revolution of 2011.” Tunisia’s transition to democracy has been a sign of hope amongst the other failures of the Arab Spring, with the quartet underpinning the Ennahda–led Islamic government’s willing cessation of power. Although peace in Tunisia is still fragile, the president of the Human Rights League, Abdessattar Ben Moussa said that winning the Nobel Peace Prize “proves that dialogue is the only way to solve a crisis, and not weapons.” (*New York Times*, 10 Oct 2015)

## SCIENCE AND TECHNOLOGY

➢ Cambodia’s traditional diet of rice and fish is low in iron, which leaves many people at risk of iron deficiency and hence anaemia. Christopher Charles, a medical student at McMaster University in Canada, has designed a simple solution that could provide 75% of daily iron needs. He designed a small iron fish, to be placed in a litre of water and boiled for 10 minutes with some citrus to aid iron absorption. The resulting iron–rich water can be used for drinking or cookery. The idea was inspired by iron leaching into food from iron–based cooking pans – the fish is a substitute for these pans which are unsuitable for most Cambodian people. Christopher’s iron fish won the Grand Prix for product design at France’s Cannes Lions International Festival of Creativity. (*slate.com*, 29 June 2015)

➢ According to a study published in *The Lancet Psychiatry*, daily tobacco smokers may be at increased risk of psychosis. It found that 57% of people with their first episode of psychosis are smokers – nearly 3 times higher than the general population. This high rate has long been recognised, but it was generally believed that people used tobacco to alleviate the distressing symptoms of psychosis and possibly the side–effects of anti–psychotic drugs. However, tobacco may have a causal effect, which is consistent with excess dopamine causing psychotic illnesses – and nicotine exposure increases dopamine levels. The exact relationship is more subtle, as people tend to inherit vulnerability to developing schizophrenia, and other factors will increase the risk. Prof Michael Owen of the Institute of Psychological Medicine and Clinical Neurosciences at Cardiff University hopes that further genetic research will establish the relationship, noting that “the fact is that it is very hard to prove causation without a randomised trial, but there are plenty of good reasons already for targeting public health measures very energetically at the mentally ill.” (*The Guardian*, 10 July 2015)

➢ Results from a UK study published in *The Lancet Neurology* suggests that the risk of developing dementia is now declining. This balances the increasing number of people living into their 80s and 90s, so that the overall number of people with dementia remains reasonably stable. This potential decline may be caused by improved physical health–linked to better nutrition and less infectious diseases; improved cardiovascular health which has a protective effect on brain health; and increasing levels of mental stimulation. Interventions to promote general health may be more effective that wide–scale screening for dementia, partly because existing tests can be unreliable and add little to treatment options. However, the UK’s Alzheimer Society highlight that the results could be overturned by other trends, eg, increasing rates of obesity and diabetes. (*New Scientist*, 21 Aug 2015)

➢ William C Campbell, Satoshi Ōmura and Youyou Tu won the 2015 Nobel Prize in Physiology or Medicine for their work in developing therapies against parasitic infections. Campbell and Ōmura discovered a class of compounds (avermectins) which kill parasitic roundworms which cause infections such as river blindness and lympathic filariasis. Tu, the first China–based scientist to win a science Nobel, developed the anti–malarial drug artemisinin. Stephen Ward of the Liverpool School of Tropical Medicine says that these awards highlight the global acceptance of the importance of parasitic infections, and neglected tropical diseases, noting that artemisinin has saved “millions” of lives, and that avermectins have protected millions from parasitic roundworms. Each year, Merck donates 270 million treatments of avermectin. (*Nature*, 5 Oct 2015)

➢ As Swaziland is poised to become the first malaria–free country in sub–Saharan Africa, the world should aim beyond controlling infectious diseases, and look towards the eradication of certain diseases. Measles, mumps, rubella, filariasis, pork tapeworm, malaria and hepatitis C are plausible targets; eliminating them would save 1.2 million lives each year–and transform many millions more. Mass vaccination campaigns can decrease disease levels, but parasites evolve resistance, and funding may dry up when political attention turns elsewhere–causing diseases to bounce back. Improved communications that support monitoring disease outbreaks; better medical technology in the form of drug innovations; and genetic engineering’s potential all make eradication more achievable. The emergence of HIV/AIDS and Ebola has also galvanised political will and led to improved health infrastructure. Candidates for eradication will change over time; and HIV, one of the biggest prizes, shares with smallpox the vulnerability of human–only hosts and the inability to survive independently. (*The Economist*, 10 Oct 2015)

